# Reverse Engineering and 3D Printing of Medical Devices for Drug Delivery and Drug-Embedded Anatomic Implants

**DOI:** 10.3390/polym15214306

**Published:** 2023-11-02

**Authors:** Anusha Elumalai, Yash Nayak, Aravinda K. Ganapathy, David Chen, Karthik Tappa, Udayabhanu Jammalamadaka, Grace Bishop, David H. Ballard

**Affiliations:** 1 3D Printing Lab, Mallinckrodt Institute of Radiology, Washington University School of Medicine, St. Louis, MO 63110, USA; anushae@wustl.edu (A.E.); nayakyr@wustl.edu (Y.N.); aganapathy@wustl.edu (A.K.G.); david.chen@wustl.edu (D.C.); 2 Department of Breast Imaging, Division of Diagnostic Imaging, The University of Texas, 7000 Fannin Street, Houston, TX 77030, USA; kktappa@mdanderson.org; 3 Biomaterials Lab, Rice University, 6100 Main St., Houston, TX 77005, USA; umj1@rice.edu; 4 Mallinckrodt Institute of Radiology, Washington University School of Medicine, St. Louis, MO 63110, USA; bishop.g@wustl.edu

**Keywords:** 3D printing, drug delivery, medical applications, reverse engineering, implants, healthcare

## Abstract

In recent years, 3D printing (3DP) has advanced traditional medical treatments. This review explores the fusion of reverse engineering and 3D printing of medical implants, with a specific focus on drug delivery applications. The potential for 3D printing technology to create patient-specific implants and intricate anatomical models is discussed, along with its ability to address challenges in medical treatment. The article summarizes the current landscape, challenges, benefits, and emerging trends of using 3D-printed formulations for medical implantation and drug delivery purposes.

## 1. Introduction

Reverse engineering is a fast and cost-effective technique for crafting functional or nonfunctional replicas of existing objects. In the context of rehabilitation, the process of reverse engineering can be delineated into a sequence of coherent stages facilitated through semi-automated means:

- Acquisition of 3D geometrical data: This initial phase involves digitally capturing 3D geometric information. These data can be directly gleaned from the patient or extracted from their medical records. Advanced imaging techniques such as computed tomography (CT) or magnetic resonance imaging (MRI) play a pivotal role in this stage, precisely capturing patient-specific anatomical details.
- Modification and Adaptation: Following data acquisition, the subsequent step entails modification and adaptation procedures. This stage serves as a bridge between raw data and tailored solutions. It involves fine-tuning the acquired data to align with the unique requirements specific to the patient.
- Creation of a 3D model or final product: With refined data, the process advances to create a 3D model or the ultimate product. Employing cutting-edge 3D printing technology, these models or products are realized. This stage further encompasses meticulous control over various aspects, including material attributes, shape, dimensions, and, most importantly, patient comfort.

The confluence of these stages generates a streamlined and efficient process, enabling the realization of patient-specific solutions in targeted drug delivery options. This approach, reinforced by reverse engineering principles, showcases the potential to reshape the redesign of personalized medical interventions for more effective drug delivery for localized regions in cases of chemotherapeutic drugs or drugs with severe side effects.

## 2. Medical 3D Printing

Originating in the 1980s from the automotive and aerospace sectors, 3DP has experienced a transformative journey. While its roots lie in these industries, recent strides in the medical domain have ushered in a wave of revolutionary possibilities, particularly within the pharmaceutical sector, where the concept of personalized medicine has found fertile ground for growth [1]. This section provides a concise exploration of the interplay between 3DP and medicine. Within this discourse, we shall delve into the merits and applications of 3DP, casting light upon its profound implications for medical practices and systems. By tracing its evolution, we unravel how 3DP has transcended its initial industrial confines, imprinting an indelible mark on the landscape of medical innovation. As we navigate this overview, the focal point remains on the symbiotic relationship between 3DP and the dynamic scope of drug delivery. Herein the fusion of technology and healthcare catalyzes a paradigm shift. We aim to illuminate the manifold benefits that 3DP brings to the patient and targeted drug delivery, from personalized medical solutions to the broader range of medical systems.

Three modalities of 3DP have been explored for producing pharmaceutical formulations: laser-based writing systems, printing-based inkjet systems, and nozzle-based deposition systems [2]. Laser-based 3DP, such as stereolithography (SLA), involved the use of an ultraviolet laser beam to cure resin into a crosslinked polymer [3] and was the first style of 3DP technology. Resolution varies depending on the printing technique used (SLA vs. digital light procession (DLP) or multijet printing (MJP)). A key advantage to laser-based systems includes the ability to print large-size models and the increased resolution [4,5]. However, a key disadvantage is the lack of FDA-approved resins and material properties, which can result in brittle and unstable prints [4,5,6]. Material extrusion printers include fused-deposition modeling (FDM) or fused filament fabrication (FFF) printers, although there is no functional difference between the two [7]. The advantages of simple extrusion-based printing involve lower cost of materials and ease of use, while the disadvantages include lower resolution and quality of prints [3]. Over 80% of published data for 3DP applications utilized extrusion-based printers [8]. Inkjet systems were originally introduced for office-based applications but have crossed into the domain of 3DP due to their high precision; they dispense small volumes of liquid materials onto a substrate [9].

Polyjet or multijet printing (MJP) offers flexibility in producing models of varying density and color, but the main disadvantage is the high cost [10]. The first FDA-approved oral drug delivery system, “Spritam [4,5]” was developed using the drop-on-solid technique, which involves the deposition of liquid onto a powder bed to form a solid structure [4]. Three modalities of 3DP have been explored for producing pharmaceutical formulations: laser-based writing systems, printing-based inkjet systems, and nozzle-based deposition systems [2]. Laser-based 3DP, such as stereolithography (SLA), involved the use of an ultraviolet laser beam to cure resin into crosslinked polymer [3] and was the first style of 3DP technology [4]. Resolution varies depending on the printing technique used (SLA vs. Digital Light Procession (DLP) or multijet printing (MJP). A key advantage to laser-based systems includes the ability to print large-size models and the increased resolution [4,5]. However, a key disadvantage is the lack of FDA-approved resins and material properties, which can result in brittle and unstable prints [5,6]. Material extrusion printers include fused-deposition modeling (FDM) or fused filament fabrication (FFF) printers, although there is no functional difference between the two [7]. The advantages of simple extrusion-based printing involve lower cost of materials and ease of use, while the disadvantages include lower resolution and quality of prints [3]. Over 80% of published data for 3DP applications utilized extrusion-based printers [8]. Inkjet systems were originally introduced for office-based applications but have trespassed into the domain of 3DP due to their high precision; they dispense small volumes of liquid materials onto a substrate [9]. Polyjet or multijet printing (MJP) offers flexibility in producing models of varying density and color, but the main disadvantage is the high cost [10]. The first FDA-approved oral drug delivery system, “Spritam [5,11]” was developed using the drop-on-solid technique, which involves the deposition of liquid onto a powder bed to form a solid structure [11].

The first FDA-approved oral drug delivery system, “Spritam [4,5]” was developed using the drop-on-solid technique, which involves the deposition of liquid onto a powder bed to form a solid structure [4]. Aprecia Pharmaceuticals Company was established in 2003 with the goal of achieving higher production rates for pharmaceutical applications of powder-liquid 3D printing. They became the exclusive licensee for this technology and focused on improving machine designs for faster production. Their initial success was with “fast melt” dosage forms known as ZipDose technology [12].

A high dose cardiometabolic drug demonstrated success, disintegrating in seconds with positive stability and bioavailability. Aprecia later shifted its focus to central nervous system drugs, particularly levetiracetam, leading to the approval of SPRITAM, marking the first regulatory approval for a pharmaceutical product manufactured using 3D printing. The approval of SPRITAM also signifies a renewed interest in applying 3D printing and other additive manufacturing techniques to pharmaceuticals and life sciences over the last decade.

Among the various other methods of 3D printing, FDM stands out as the most popular. This technique utilizes heat to liquefy a filament made of FDA-approved thermostatic material PLA. The molten filament is guided through an extruder and meticulously deposited layer-by-layer to create three-dimensional prints. FDM 3D printing proves to be an exceptional choice for producing functional prototypes and end-use parts due to its capability for precision and versatility. FDM has gained popularity due to some advantages over other printing methods. Some of the benefits include:

- Diverse filament options: FDM offers a broad selection of filament materials providing flexibility in design and functionality.
- Speed and efficiency: This technology allows for rapid production ensuring a quick turnaround time for finished parts and assemblies.
- Cost-effectiveness: It is more affordable compared to other 3D printing techniques, making it budget-friendly choice for various applications.
- Durability: It produces robust and sturdy models, making it ideal for parts intended for harsh environments or heavy use.
- Complex geometries: It excels in handling projects involving large pieces and intricate designs, often at a lower cost to size ratio than alternative methods.
- Precision and consistency: This method enables high precision production with consistent and repeatable results, ensuring quality across multiple iterations.
- Toolless manufacturing: All that is required to create a part is a 3D printer, eliminating the need for specialized tools or molds.
- Environmentally friendly: This technique is eco-friendly emphasizing sustainable practices in manufacturing processes.

Despite its numerous advantages, FDM does has some drawbacks. One notable limitation is that FDM-printed parts tend to exhibit lower resolution and rougher surface finishes when compared to those crafted through techniques such as SLA and some other 3D printing methods.

3DP works similarly as various functional materials are deposited on polymeric substrates, representing an intersection between extrusion-based printers and laser-based systems. UV light is used to harden the polymer after it is extruded onto the substrate [11]. Furthermore, the 3D printing process affects both the drugs and the polymers used in the process. The major drawback in employing 3DP technologies is the use of UV radiation sources or high temperatures during the 3DP process which can lead to degradation of any embedded drugs. The polymers undergo physical–chemical changes during the printing process. For instance, FDM printing causes melting and solidification causing changes in crystallinity. These changes in crystallinity in turn affect degradation, solubility and stability of the 3D-printed medical device.

### 2.1. Common Reverse Additive Manufacturing Techniques Used for Drug Delivery Applications

Additive manufacturing techniques enable customizability, especially in the production of personalized medicines with precise dosages allowing high control over release profiles and delivery locations. Commonly used 3DP techniques used for drug delivery applications include FDM, SLA, powder bed fusion (PBF), and inkjet printing.

Fused deposition modeling is a commonly used 3DP technique in the pharmaceutical and medical sectors. In FDM, a thermoplastic filament is melted and extruded layer-by-layer to create the desired structure. FDM offers several advantages for drug delivery applications, such as the ability to create complex geometries, control the porosity of the structure, and incorporate drugs into the filaments.

One of the most popular applications of FDM in drug delivery is the creation of drug-loaded filaments. Pharmaceuticals are first embedded into the polymer matrix and extruded as filaments of the required dimensions, which are then used in the 3DP machine to create a prototype or drug delivery device that can be orally used as tablets, implanted, or injected into the body. The drug concentration and release can be controlled by adjusting the amount of drug to be loaded in the polymer matrix as well as changing the porosity of the model that will be 3D printed.

Scoutaris et al. 3D-printed indomethacin-loaded chewable tablets using polyethylene glycol (PEG) polymer filaments using the FDM technique. For enhanced acceptability of the tablets by pediatric patients, formulations were fabricated in the form of variable shapes, including a lion, heart, bottle, ring, and bear [13]. Sadia et al. designed perforated channels within the caplets to enhance drug release rates and used FDM techniques to 3D-print hydrochlorothiazide caplets for evaluation [14]. Similarly, Oblom et al. used non-identical cellulose-based polymers including hydroxypropyl methyl cellulose (HPMC), hydroxypropyl cellulose (HPC) and Eudragit loaded with the isoniazid drug to fabricate dosages that treat and prevent latent tuberculosis. Their research has shown that altering printing parameters including tablet size and infill ratios can allow personalization of the tablets [15].

Stereolithography: SLA is a 3DP technique that uses a laser to solidify a liquid resin layer-by-layer to create a 3D structure. SLA has emerged as a promising technique for drug delivery applications due to its ability to create complex and precise structures with high resolution. Xu et al. formulated ibuprofen-loaded mini-sized pellets for oral administrative applications using SLA technology. They have used polyethylene glycol diacrylate (PEGDA) as monomer and diphenyl (2,4,6-trimethyl benzoyl) phosphine oxide (TPO) as photo initiator [16]. In a research study conducted by Robles-Martinez et al., multilayer polypills were fabricated using six drugs including paracetamol, caffeine, naproxen, chloramphenicol, prednisolone, and aspirin, in various shapes and compositions. The authors have successfully demonstrated the feasibility of SLA as an excellent 3DP technique to manufacture multi-dosage formulations [17].

SLA has also been used to create microneedle arrays for transdermal drug delivery. Economidou et al. used SLA to create microneedle arrays containing insulin in both pyramidal and spear-shaped microneedles for transdermal administration. The evaluation of these 3D-printed microneedles in a mice model showed an enhanced skin penetration compared to subcutaneous injections [18]. Xu et al. engineered solid and hollow intravesical bladder devices using elastic resins in SLA-type 3D printer. Solid devices were manufactured by direct mixing of lidocaine hydrochloride with elastic resin and 3DP solid constructs. In contrast, hollow devices were first 3D-printed with elastic resin and later loaded with lidocaine hydrochloride. Comparative evaluation among these devices showed varying drug release profiles and the authors established a novel SLA technique to fabricate localized and extended intravascular delivery devices [19].

In the PBF type of 3DP, a layer of powder is spread across the build plate and selectively melted using a laser or electron beam to create the desired shape. Additive manufacturing techniques including selective laser sintering (SLS), selective laser melting (SLM), and electron beam melting (EBM) are some of the common methods that can be categorized under PBF. Of all these techniques, SLS is the only non-metallic process that can utilize biocompatible and biodegradable material to produce 3D-printed objects. This process has a close resemblance to traditional tablet-manufacturing process. Additionally, this technique enables creating complex and porous structures with high resolutions. Due to these advantages, researchers have explored SLS technology and its assisted materials, for numerous drug delivery applications [20].

Gueche et al. utilized SLS type of PBF technique to manufacture solid oral dosage forms from copovidone and paracetamol using carbon dioxide laser sintering. Authors in this research have demonstrated that utilization of copovidone has allowed fabrication of dosage forms without additional absorbance enhancers [21]. In another study, Salmoria et al. developed intrauterine device (IUD) drug delivery systems containing female sex hormones including, progesterone and fluorouracil, utilizing SLS techniques for hormonal replacement therapy and to enhance cancer treatment at the site of tumor growth. Their studies have demonstrated that utilization of higher power laser yielded IUDs with enhanced mechanical properties [22].

In inkjet printing, droplets of drug-loaded resin are ejected/sprayed on the substrate/build platform in layer-by-layer fashion fusing with layers underneath to create an object of the desired shape. Inkjet printing has demonstrated great potential for drug delivery applications, particularly for creating precise and personalized drug delivery systems. Its ability to create complex patterns with high resolution makes it an attractive option for developing advanced drug delivery devices.

In a research study conducted by Boehm, anhydride copolymer microneedles were manufactured using SLA-type 3DP technology, where these needles were surface-coated with miconazole using inkjet technology to produce transdermal microneedles for cutaneous fungal infection treatment [23]. Similarly, Pollard et al. modified a commercially available inkjet 3D printer to surface-coat timolol maleate drug onto contact lenses. This glaucoma therapy drug was released from the contact lenses for over 3 h, which was significantly longer than the traditional eye drop usage [24].

### 2.2. Biomaterials Used in 3D Printing

#### 2.2.1. Introduction to Biomaterials:

**Figure 1:**
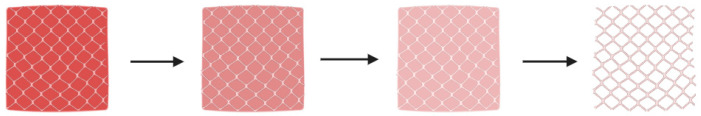
Degradability of polymers by bulk erosion over time.

Drug delivery systems are printed with different polymers and by varying methods depending on the goal of the print [23]. Polymers fall under two broad categories: biodegradable and non-biodegradable [24]. Biodegradable polymers are generally categorized as having the ability to erode into the human body over time [24]. Biodegradable polymers can be subdivided into natural and synthetic biomaterials [23,24,25]. Biodegradable natural polymers, such as gelatin, alginate, and collagen, come from biological sources, making them useful for fabrication of biodevices due to their compatibility with native proteins of the human body [25,26]. Furthermore, natural polymers can crosslink when exposed to the appropriate stimuli, making them useful for creating microgels and hydrogels [25,26]. Unfortunately, the stimuli required to induce crosslinking can be cytotoxic [23]. On the other hand, biodegradable synthetic polymers, such as poly (L-glycolic acid) (PGA), poly (L-lactic acid (PLA), poly (D, L-lactic-co-glycolic acid (PLGA) and poly (ε-caprolactone) (PCL), are frequently utilized to fabricate drug delivery systems due to their low cost and widespread FDA approval [25,27]. Degradable polymers degrade via bulk erosion as seen in Figure 1.

However, these compounds have the drawback of being less biocompatible [25]. The second broad category of polymers includes non-biodegradable compounds such as PEG and ethylene vinyl acetate [24]. Unlike biodegradable polymers, these compounds remain structurally intact during their life cycle. 3DP can use properties that vary across different polymers, such as porosity, hydrophobicity, and drug release, to engineer customized microfluidic drug delivery devices [24]. Drug delivery systems are printed with different polymers and by varying methods depending on the goal of the print [25]. Polymers fall under two broad categories: biodegradable and non-biodegradable [26]. Biodegradable polymers are generally categorized as having the ability to erode into the human body over time [26]. Biodegradable polymers can be subdivided into natural and synthetic biomaterials [25,26,27]. Biodegradable natural polymers, such as gelatin, alginate, and collagen, come from biological sources, making them useful for fabrication of biodevices due to their compatibility with native proteins of the human body [25,26]. Furthermore, natural polymers can crosslink when exposed to the appropriate stimuli, making them useful for creating microgels and hydrogels [27,28]. Unfortunately, the stimuli required to induce crosslinking can be cytotoxic [23]. On the other hand, biodegradable synthetic polymers, such as Polyglycolic acid, Polylactic Acid, and Polycaprolactone, are frequently utilized to fabricate drug delivery systems due to their low cost and widespread FDA approval [27,29]. However, these compounds have the drawback of being less biocompatible [27]. The second broad category of polymers includes non-biodegradable compounds such as Polyethylene Glycol and Ethylene Vinyl Acetate [26]. Unlike biodegradable polymers, these compounds remain structurally intact during their life cycle. 3DP can use properties that vary across different polymers, such as porosity, hydrophobicity, and drug release, to engineer customized microfluidic drug delivery devices [26].

#### 2.2.2. Physical, Chemical and Biological Properties of Biomaterials

Most synthetic biopolymers such as PLA, PGA, PLGA, and PCL are 3D-printed using the FDM technique. This method requires the polymers to be heated to their melt extrusion temperatures. These temperatures are higher than the melting temperature. Depending on the composition of the polymer, the melt extrusion temperatures range from 90–220 °C. Viscoelastic properties of these polymers are highly dependent on temperature and composition. Drugs or bioactive agents that are thermally stable can be used with these polymers. These polymers are also used in SLS-based 3DP; fine powders of the polymer are melted using high energy lasers [30].

The other biomaterials used in extrusion or FDM printing include synthetics (PEG, poloxamers, etc.) and natural polymers (alginate, collagen, gelatin, decellularized extracellular matrix, etc.). These biomaterials undergo crosslinking when exposed to suitable external stimuli. The crosslinking stimuli can be physical (light, heat) and chemical (counter ions) stimuli. The mechanical properties of the biomaterials improve after crosslinking as polymers are held in place by covalent and ionic bonds. Mechanical properties of biomaterials are very important as they determine how the material maintains shape, retains architecture, and enables easy handling of the 3D-printed structures [30].

Another important property of biomaterials that needs to be considered is the degradation process, products of degradation and route of elimination. Polymers primarily undergo bulk erosion or molecular degradation. In the case of bulk erosion, the scaffolds undergo hydrolysis at random ester bonds and undergo further hydrolysis to release monomers into the tissue. Depending on the type of polymer used, these monomers could be lactic acid, glycolic acid, fumarate etc. These monomers, components of physiological processes such as the Krebs cycle, are eliminated through the lungs [31]. Bioceramics are a class of biomaterials used in fabrication of implants that are used in orthopedic applications. These bioceramics are resorbed by surrounding cells to promote new tissue development [32].

Biomaterials used in vat polymerization-based 3DP methods such as SLA and DLP are modified by addition of acrylate groups. Commonly used biomaterials include gelatin methacrylate (GelMA), polyethylene glycol diacrylate (PEGDA), and hyaluronic acid methacrylate (HAMA), etc. Methacrylate and diacrylate polymers in the presence of photointitators such as Irgacure, LAP, etc. undergo free radical polymerization when exposed to UV or visible light to enable crosslinking. These photoinitiators are cytotoxic and hence should be used with caution. The biomaterials used in vat polymerization methods have lower viscosity on the millipascal-second order. The resolution of the objects printed using vat polymerization depends on the energy and point size of the light source [33].

In binder jet-based 3DP, two biomaterials are used—one in the powder form and the other in the liquid form. The packing density, particle size and flowability of the powder are important properties. Printability and resolution of the printed objects depends on the size of the powder particles. Layer thickness in binder jet printing is higher than the particle size (of the powder) and ranges from 15–300 μm. A few of the commonly used biomaterials in binder jet 3DP include titanium and its alloys, cobalt–chromium alloys, calcium phosphate salts, polymers, and composites. The binders provide mechanical stability to the printed object by gluing the powder particles together. Low-viscosity materials such as PVA solution, water, phosphoric acid, etc., are used as binders for biomedical applications [34].

### 2.3. Research Relating Drug Release Properties When 3D Printing Is Used

#### 2.3.1. Targeted Approach

**Figure 2:**
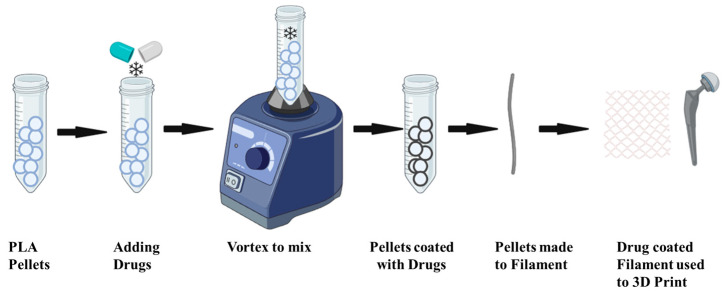
Applying a drug coating to PLA pellets to create a filament for use in 3D printing.

Drug delivery via implants can be a very effective manner of drug delivery, benefitting those patients who need long-term treatments. The aim of using reverse-engineered 3DP implants is to create a patient-specific, reproducible loading for effective doses of drugs by using MRI/CT scans. Loading can be accomplished via incorporation into the printing process or during the printing process itself. When using FDM printing, drugs can be incorporated in making the filaments as seen in Figure 2.

The drugs are coated to the pellets using oil casting [33,34,35]. The drug mixes and distributes in the filament homogenously. In the inkjet method, the drug is incorporated into the powder bed or binder solution [35]. For DLP the drugs are incorporated into the printed matrix by dissolving or suspending them in liquid photopolymers [36]. Drugs can also be coated post-printing to the finished 3D-printed implants. The drugs embedded in printing must withstand high temperatures and other printing processes. Since the drug is incorporated in the implants, long-term drug release is conceivable using this method. Using 3DP for drug-eluting implants can play an important role in organ printing, tissue engineering, and making individual molds for medical and pharmaceutical needs [37,38,39]. Drug-eluting 3DP can make stents, catheters, bone screws, gynecological devices, and antitumor devices. The risk of infection is high when a foreign object is inserted in the body. With 3D-printed implants, such risks can be potentially manageable by embedding them with antimicrobial drugs. Table 1 shows a summary of some drugs used via various 3D printing techniques.

Sandler et al. [40], showed successful incorporation of nitrofurantoin in PLA filaments. Weisman et al. [41] confirmed such findings by showing the inhibitory effect of 3D-printed discs containing drugs such as gentamicin sulphate and methotrexate on *Escherichia coli* (*E*. *coli)* and a decrease in number of osteosarcoma cells respectively. The process of loading the filament with drugs and printing the 3D disc did not reduce the effectiveness of the drugs. After post-processing, Boyer et al. [42] showed that complex structures such as 3D-printed mesh and vascular Y-stents had a high visibility when CT-scanned and even had antibacterial effects. Stents supporting palate and lip surgery are being researched by Mills et al. [43] and Boyer et al. [42].

**Figure 3:**
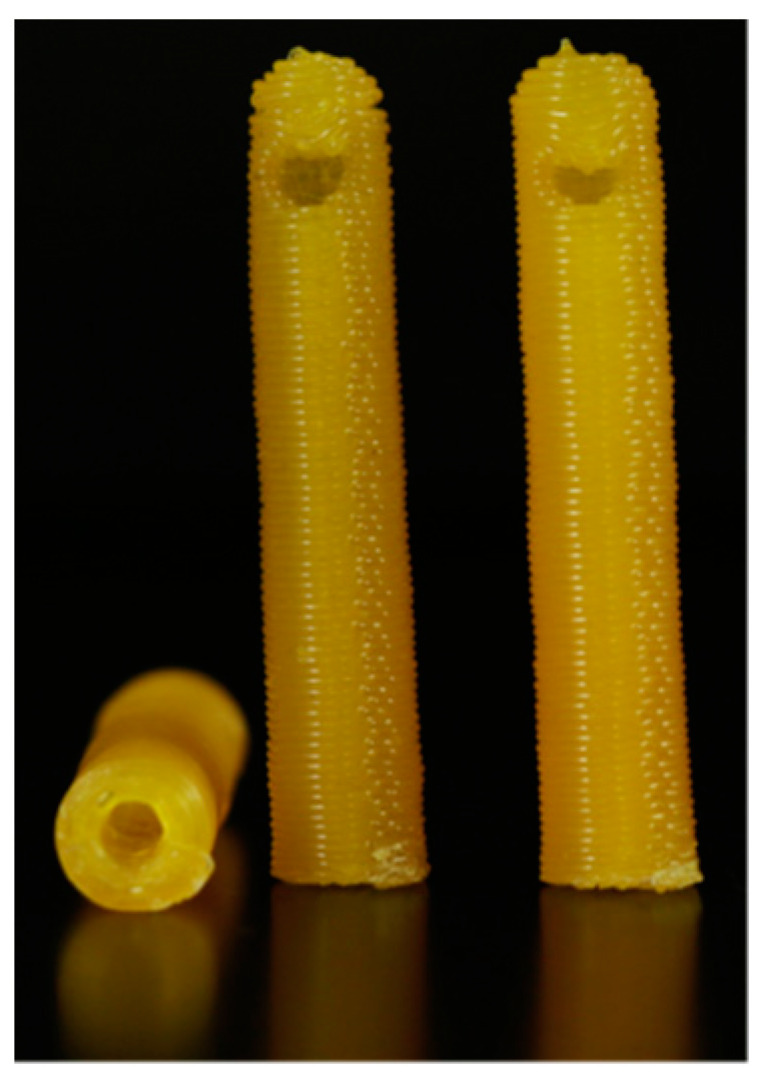
Photograph of a 3D-printed methotrexate-embedded catheter. Reproduced from Weisman et al. [44].

**Figure 4:**
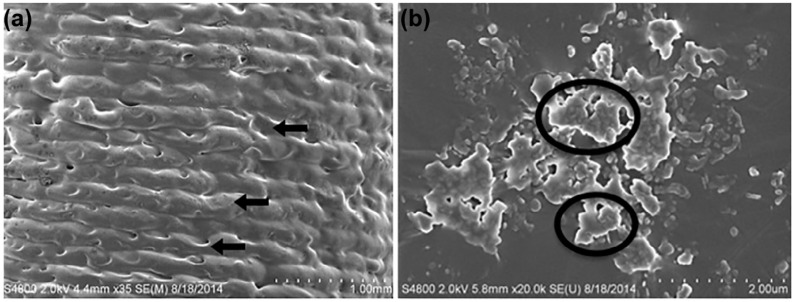
Images of gentamicin-embedded 3D-printed catheters captured by scanning electron microscope (**a**,**b**). In Figure 4a, the arrows indicate layer by layer process. In Figure 4b, the amorphous effect seen (circled) shows the incorporation of Gentamicin. Reproduced from Weisman et al. [44].

3D-printed catheters embedded with antibiotics and chemotherapeutics using FDM technology were shown to have an initial burst of release followed by a steady release rate for five days by Weisman et al. [41]. Figure 3 shows the model of a drug-embedded catheter and Figure 4 shows the surface topography of a gentamicin-embedded catheter printed by Weisman et al. [44]. Longer antibacterial effects can be anticipated by changing the concentration of drug loaded in the filaments.

One current example of drug-releasing devices is the intrauterine device (IUD), which provides long-term contraception with localized hormone delivery [45]. One issue with IUDs is the difference in shape and size of the endometrial cavity between individual women. Using 3DP could assist in making well-fitted IUDs to overcome these dimensional challenges. However, special attention must be paid to the specific materials used in the 3DP process, as shown by Genina et al. [46]. Adhesion, polarity, flexibility, crystallinity, and melting point may impact the print’s quality and durability.

Materials such as flexible TPU have been used to print bacteriostatic vaginal meshes. These meshes were loaded with doses of levofloxacin for treating stress urinary inconsistence and pelvic organ prolapse [47]. Zhao et al. [48] have also applied changes in printing techniques to 3D-print cone-shaped cervical tissue implants. Here, micro and macro pores in the printed structures mimicked the tissue properties—enabling loading of anti-HPV proteins.

Research has also shown that drugs such as minocycline, gentamicin, isoniazid, rifampicin, and vancomycin, when used in 3DP implants, have been helpful in treating bone fractures and other injuries [49,50,51,52]. Along with the reparative drugs, glucocorticoids such as prednisolone and dexamethasone have been used successfully to make scaffolds [53,54]. Wu et al. [55] used inkjet technology for 3DP multidrug implants for treating tuberculosis extending into bone. Furthermore, Poudel et al. [56] explored the use of laser powder bed fusion to create 3D-printed orthopedic implants from surgical grade 316L stainless steel. The implants are coated with PLGA and loaded with gentamicin to provide sustained antibiotic release, combating post-surgical infections and enhancing cell adhesion, with proven efficacy against common pathogens such as *S. aureus* and *S. epidermidis*.

Surgical meshes are classically used to treat hernias [57]. 3DP techniques to print meshes loaded with antibacterial, anti-inflammatory drugs, and contrast agent have all appeared in the literature [57,58,59]. Visibility via CT imaging was performed by Ballard et al. [59]. Hollander et al. [58] developed a printed mesh of medical grade liquid silicone rubber with different pore sizes. It was embedded with prednisolone and showed promising results.

Utilizing reverse-engineered three-dimensional printing (3DP) technology for the localized delivery of antibacterial agents at the implant site presents significant potential in mitigating infection and promoting implant longevity throughout the recovery period. In the context of cancer treatment, numerous antitumor medications exhibit low solubility in aqueous solutions [60]. Current research explores the localized administration of chemotherapeutic agents such as fluorouracil, methotrexate, and cytoxan [50,54,55], utilizing 3DP techniques to target malignant cells directly [61,62]. Factors including the choice of material, infill ratio, design, dimensions, printing methodology, and drug-release characteristics influence the release rate of these drugs.

#### 2.3.2. Current Applications of 3D Printing in Drug Delivery

Implantable drug delivery devices: Several commercially available implantable drug delivery devices have been developed to provide controlled drug release over an extended period. Some examples include:

- Infuse Bone Graft: This implantable device, manufactured by Medtronic, delivers recombinant human bone morphogenetic protein-2 (rhBMP-2) to promote bone growth in spinal fusion procedures. 3D printing could create patient-specific implants with optimized geometry and drug release profiles, improving surgical outcomes and reducing complications.
- Zoladex: A biodegradable implant developed by AstraZeneca for treating prostate cancer and certain gynecological disorders. It releases the drug goserelin over time, which helps regulate hormone levels. 3D printing could enable the development of implants with customizable drug release rates and more precise control over hormone regulation.
- Norplant: A subdermal contraceptive implant that releases the hormone levonorgestrel over an extended period. It has been replaced by newer systems such as Nexplanon and Implanon. 3D printing could be used to develop patient-specific implants that deliver the optimal drug dose based on individual patient needs and characteristics, potentially reducing side effects and improving efficacy.
- Vitrasert: An ocular implant used to treat cytomegalovirus retinitis in patients with AIDS. The implant releases the antiviral drug ganciclovir over an extended period. 3D printing could be used to develop customized ocular implants that conform better to individual patient anatomy, improving drug delivery and reducing complications.
- Probuphine is a subdermal implant that delivers buprenorphine to treat opioid dependence. Titan Pharmaceuticals developed the implant, which provides continuous drug release for up to six months. 3D printing could create personalized implants that optimize drug release based on individual patient needs, potentially improving treatment outcomes, and reducing relapse rates.

3D printing technologies can potentially improve upon these implantable drug delivery systems by offering:

- Customization: 3D printing enables the creation of patient-specific implants that match individual patient anatomy and clinical needs, potentially improving treatment outcomes and reducing complications.
- Precision: 3D printing allows for precise control over implant geometry, material properties, and drug release profiles, which could lead to more effective and safer drug delivery.
- Complex geometries and multi-component systems: 3D printing can produce implants with intricate structures and multiple drugs, allowing for more sophisticated drug delivery strategies.
- Rapid prototyping and production: 3D printing technologies enable faster development and production of implantable drug delivery devices, potentially speeding up bringing new devices to market.

#### 2.3.3. Drug Release Rate

Drug release rate refers to the speed at which drugs become pharmacologically active [25]. The main types of drug-release include immediate release, delayed, sustained, and controlled release [25]. An immediate-release drug delivery system aims for rapid onset of drug activation post administration. To minimize delay in drug action, the drug must have high solubility and permeability to cross mucosal membranes for absorption [25]. Okwuosa et al. notes the development of immediate-release tablets with a disintegration time of less than 15 min created via low-temperature fused deposition modeling (FDM) 3D-printing of polyvinylpyrrolidone [63]. Bhatt et al. further notes the utility of combining hot-melt extrusion (HME) with FDM 3D-printing to create immediate-release olanzapine tablets with a mean disintegration time of 63.33 s [64].

A sustained release drug-delivery system consistently delivers a drug overtime to overcome rapid metabolization or elimination by the body [25]. Giri et al. describes the use of selective laser sintering (SLS) 3D printing to design tablets with a Kollidon SR (KSR) matrix [65]. Their tablets could gradually deliver acetaminophen over a 12-h period rather than in a single burst [65]. Additionally, Wu et al. describes using 3D-printed sustained-release scaffolds composed of bioactive glass, alginate, and gelatin [66]. When co-printed with naringin and calcitonin gene-related peptide, their scaffold delivered active drug for 21 days with no initial burst release [66].

## 3. Limitations

Research in developing 3DP is on the way to more promising improvements in the medical sector. Currently, many challenges are limiting the use of this application such as biocompatible material, quality control, and regulatory acceptance. There are many 3DP technologies available, which generates a need to test and compare the different parameters and optimize the drug dosage with efficacy and stability. The major drawback in employing 3DP technologies is the use of UV radiation sources or high temperatures during the 3DP process which can lead to degradation of the embedded drugs. These technologies also require a post-processing step to retain the mechanical properties. Some steps include removal of support, washing of excessive resin and then curing (in the case of SLA and DLP printing), drying (in the case of inkjet printing), and heat step (in the case of powder-based printing). The use of reverse 3DP may make the delivery patient-specific but not necessarily appealing to the patient. The final product may have uneven or rough surfaces. Although 3DP has found extensive use in the medical field, this technology for delivering drugs is still in development, with most of the current research limited to in-vitro studies. Additional studies will be required. There are FDA-approved guidelines for using 3DP medical devices, but there is an absence of official guidelines for dosage delivery. Considering the need for patient-specific manufacturing, regulated specifications should be established for quality control. These are important considerations before 3DP can become viable for mass production and usage.

## 4. Prospects and Research Directions

Despite its potential, 3DP confronts several challenges to mainstream drug delivery methods. The technology’s capabilities are not entirely on par with traditional conventional approaches. As we peer into the future, particular areas of concern emerge, primarily centered around the resolution and printing speed. The impending evolution must strive to balance minimal energy consumption and low production costs, thereby enhancing the economic viability of this technology. At a micro- and nanoscale level the clarity and precision of prints necessitate marked improvement. Developing novel materials exhibiting high biocompatibility over extended durations becomes critical. In addition, a notable aspiration for the field is the effective bio-printing of tissues and organs, ensuring in vivo functionality that can seamlessly integrate with the living systems they are intended for.

These challenges, while formidable, present the impetus for ongoing research and innovation in the reverse 3D-printing arena. Addressing these concerns would pave the way for this technology to find its place alongside and surpass traditional approaches in drug delivery and medical advancement.

## 5. Conclusions

In recent decades, healthcare has witnessed a remarkable transformation with the emergence of reverse engineering 3D printing. This innovative technology has demonstrated its potential to surmount the limitations encountered by traditional medical treatments. With more than a decade of dedicated research and exploration, 3DP has garnered substantial attention for its ability to address the challenges in medical treatment by creating patient-specific implants, advanced medical devices, and intricate anatomical models. One particularly intriguing avenue of investigation within this field is the reverse engineering of 3D-printed formulations, a process that holds promise for many medical applications.

This review article aims to delve into the current landscape of reverse engineering within 3D printing technology, specifically in medical applications for drug delivery. By examining the convergence of cutting-edge 3DP technologies and the demands of the healthcare sector, we endeavor to shed light on the potential benefits, challenges, and emerging trends in developing 3D-printed formulations for medical purposes. Through this exploration, we seek to contribute to a comprehensive understanding of the transformative impact that 3D printing holds for the future of healthcare.

An emerging frontier, reverse-engineered 3DP introduces an inventive approach to drug delivery, harnessing the potential of tailored dosing strategies and bespoke anatomical conformity. By propelling this field forward, further breakthroughs promise to substantially enhance treatment efficacy, bolster patient adherence, and ultimately advance overall health outcomes.

As 3D printing progresses, its role in medicine is poised to expand significantly. Envisioned as a cornerstone of the future healthcare landscape, it is set to lay the foundation for ingenious, patient-centered, and economically viable medical solutions.

## Figures and Tables

**Table 1:** Summary of novel drug delivery applications attainable via biomaterials and 3D-printing.

Biomaterial	Printing Method	Drug Delivery Application	Reference(s)
Polyethylene Glycol Polymer (PEG)	FDM	Indomethacin loaded chewable tablets shaped as animals to appeal to pediatric patients	[13]
Cellulose-Based (HPMC, HPC, Eudragit)	FDM	Isoniazid loaded drugs to treat tuberculosis	[15]
Polyethylene Glycol Diacrylate (PEGDA)	SLA	Ibuprofen-loaded mini-sized pellets for enhanced PO intake	[16]
Polyethylene Glycol Diacrylate (PEGda)	SLA	Personalized Polypills (multiple drugs in one drug product) to reduce patient non-adherence	[17]
Dental SG Resin	SLA	Microneedle arrays for transdermal insulin delivery	[18]
Copovidone	SLS (PBF)	Solid dosage forms without need for additional absorbance enhancers	[21]
High Density Polyethylene (HDPE)	SLS (PBF)	Intrauterine (IUD) drug delivery systems containing female sex hormones	[22]
Poly (methyl vinyl ether-co-maleic anhydride))	Inkjet (SLA)	Transdermal microneedles for cutaneous fungal infection treatment	[23]
Timolol-loaded Ink	Inkjet with Near Infrared Spectroscopy (NIR)	Extended release of glaucoma therapy drug (timolol maleate) from contact lenses	[24]
